# Duplication and transcriptional divergence of three Kunitz protease inhibitor genes that modulate insect and pathogen defenses in tea plant (*Camellia sinensis*)

**DOI:** 10.1038/s41438-019-0208-5

**Published:** 2019-11-15

**Authors:** Junyan Zhu, Yaxian He, Xiaomei Yan, Lu Liu, Rui Guo, Xiaobo Xia, Daojie Cheng, Xiaozeng Mi, Lidiia Samarina, Shenrui Liu, Enhua Xia, Chaoling Wei

**Affiliations:** 1 State Key Laboratory of Tea Plant Biology and Utilization/Key Laboratory of Tea Biology and Processing, Ministry of Agriculture, Anhui Agricultural University, West 130 Changjiang Road, Hefei, 230036 Anhui People’s Republic of China; 2 Russian Research Institute of Floriculture and Subtropical Crops, 354002 Yana Fabritsiusa st. 2/28, Sochi, Russian Federation

**Keywords:** Proteases, Biotic

## Abstract

Kunitz protease inhibitors (KPIs) are ubiquitous in plants and act as crucial compounds in defense responses against insect attack and pathogen infection. However, the influence of gene duplication on the postdivergence of the *CsKPI* genes involved in biotic stresses in tea plant is not well known. Here, we identified three *CsKPI* genes from tea plant (*Camellia sinensis*) and characterized their expression and evolutionary patterns among plant species. We found that *CsKPI1*, *CsKPI2*, and *CsKPI3* diverged from their common ancestor 72.94 million years ago (MYA), and the tandem duplication of *CsKPI2* and *CsKPI3* occurred 26.78 MYA. An in vitro protein assay showed that the three CsKPI proteins were functional and inhibited the production of *p*-nitroanilide (PNA) from an artificial substrate. The three CsKPI-GFP fusion proteins localized to the cytoplasm. We showed that salicylic acid (SA) and transcripts of *CsKPI2* and *CsKPI3* significantly accumulated after infection with *Glomerella cingulata*. The application of exogenous SA stimulated the high expression of both *CsKPI2* and *CsKPI3* by activating *cis*-elements within their promoters. Under *Ectropis oblique* attack, *CsKPI1* expression and jasmonic acid (JA) levels were more abundant in both insect-damaged leaf tissues and undamaged neighboring leaves. The application of jasmonic acid methyl ester elicited high expression levels of *CsKPI1*, suggesting that *CsKPI1* accumulation requires JA production in tea plant. The overall findings suggest that the transcriptional divergence of KPI genes after duplication led to the specialized role of *CsKPI1* in the physiological response to insect stress; the functional conservation between *CsKPI2* and *CsKPI3* confers resistance to pathogen infection in tea plant.

## Introduction

The tea plant (*Camellia sinensis*) is an important economic woody crop that is cultivated worldwide^1^. Tea leaves are used to make tea beverages, which have a rich taste and confer health benefits^2^. The tea processing method, genetic variation in tea cultivars and environmental stresses are crucial factors that affect the quality of tea leaves^3^. To date, it is widely known that many abiotic and biotic stresses, such as drought^4^, low temperature^5^, insect attack^6^, and pathogen infection^7^, can induce a series of defense responses in tea plant.

Protease inhibitors (PIs) are present in almost all organisms, including animals, plants, and microorganisms; they play key regulatory roles in a wide range of biological processes^8,9^. They function by directly or indirectly blocking active centers of target proteases to strictly control their activities^8,10^. In vitro, N-α-benzoyl-dl-arginine-4-nitroanilide (BAPNA) can act as a trypsin substrate to catalyze the production of *p*-nitroanilide (PNA); PI proteins suppress the activity of trypsin and reduce the amount of PNA production. PIs are grouped into families and are induced in plants in response to injury or attack by insects or pathogens^11,12^. In *Arabidopsis*, serpin 1 proteins function as potent inhibitors that destabilize metacaspase-like proteins in vivo and function in the plant immune response^13^. Kunitz PIs (KPIs) are members of a subfamily of PIs involved in biotic defense; they function by repressing the digestive processes of insects and reducing fungal lesion development^9,14^. In *Pithecellobium dumosum*, PdPI3.1 and PdPI3.2 are members of the KPI family and affect the digestive enzymes of insect larvae of diverse orders^15^. A serine PI (Kunitz-type inhibitor designated PKI1) isolated from potato showed direct inhibitory activity on *Botrytis cinerea* strains as reported by Hermosa et al.^16^.

Ohno proposed a theory that gene duplication (GD) events represent the primary source of genetic novelty leading to speciation^17–20^. Subsequently, the subfunctionalization (SF) model was proposed: the shared functions between duplicated genes diverge over time, and these genes evolve into functionally distinct proteins, with each daughter gene specializing in a subset of functions of the ancestral gene^21,22^. Force et al. (1999) then proposed the duplication–degeneration–complementation (DDC) model; degenerative mutations accumulate within duplicated genes for a period of time, which then undergo functional specialization by complementary partition of ancestral functions^21,22^. The evolution of a gene family often results from polyploidization, whole-genome duplication (WGD), or tandem (linked) or segmental (unlinked) duplications; these have been frequent in the evolutionary history of flowering plants and have shaped the evolutionary trajectory of genomes and genes^3,23–25^. Tandem duplication can generate a copy of several genes within the same scaffold or chromosome; these events often include genes from the same networks or pathways and can accelerate the divergence of gene function. With the recent release of whole genome sequences, multiple gene families were found to undergo tandem GD in addition to functional differentiation; examples, include gene families such as dof, expansin, and phenylalanine ammonia-lyase^20,26,27^. However, there is limited knowledge regarding the mechanism of duplication events in the KPI gene family and their evolutionary trajectories in tea plant.

Plant hormones are complex and important signaling molecules that modulate many aspects of plant development and defense^28^. Salicylic acid (SA) is a phenolic compound that functions as a key signal in regulating disease resistance. It acts through effector-triggered immunity (ETI), thereby modulating the activities of diverse groups of defense-related genes; examples include those genes encoding PI proteins and several families of pathogenesis-related (PR) proteins^8,29^. Jasmonic acid (JA) is a well-known hormone regulator that mediates defense responses to insect attack^30,31^. Previous studies have indicated that PIs and JA are both strongly induced in leaves damaged by insect feeding, which implies that JA may induce the expression of PI genes and synergistically defend against insect attack^6,12,32^. In *Nicotiana attenuata*, JA and trypsin proteinase inhibitors are regulated by the BAK1 gene in response to herbivory^33^. Previous studies also indicate that plants can perceive events locally and transduce a defense signal to mount a broad and system-wide response to stresses (i.e., systemic acquired resistance (SAR))^34,35^. Although the SAR model proposes that SA and MeSA initiate SAR in plants, recent studies demonstrate that JA is also a crucial signal molecule in the SAR response; JA accumulates via de novo JA biosynthesis and then acts to transmit the signal and activate the expression of downstream-related defense genes^34,36^. Truman et al.^37^ proposed that genes involved in jasmonate biosynthesis are upregulated within 4 h, and JA then diffuses to undamaged neighboring leaves. SAR can be activated by foliar JA application and is abolished in mutants impaired in JA synthesis or the JA response^37^. Despite these studies exploring JA-mediated regulation of the SAR response, very little is known about whether KPIs are involved in SAR and which potential signals regulate KPIs under the SAR response.

Our study aims to determine the function of three CsKPI genes in tea plant. We initially identified these three CsKPIs from the tea plant genome and explored their evolutionary relationships with various plant species. Heterologous expression and subcellular localization were employed to characterize the functions of these CsKPIs. We further examined the expression patterns of the three CsKPIs in response to pathogen infection and insect attack. *CsKPI2* and *CsKPI3* exhibited high mRNA accumulation in response to pathogen infection, and this response may require SA induction. We observed that *CsKPI1* may be involved in SAR based on its expression, which was strongly induced both by localized insect feeding and in undamaged neighboring leaves. Promoter activity analysis suggested that *CsKPI1* is activated by JA, whereas *CsKPI2* and *CsKPI3* are activated by SA. Our results improve the understanding of the diversified functions of CsKPIs resulting from duplication in tea plant and highlight the underlying roles of CsKPIs in the SAR response.

## Results

### Identification of tea *CsKPIs* and evolution analysis in plant species

**Fig. 1:**
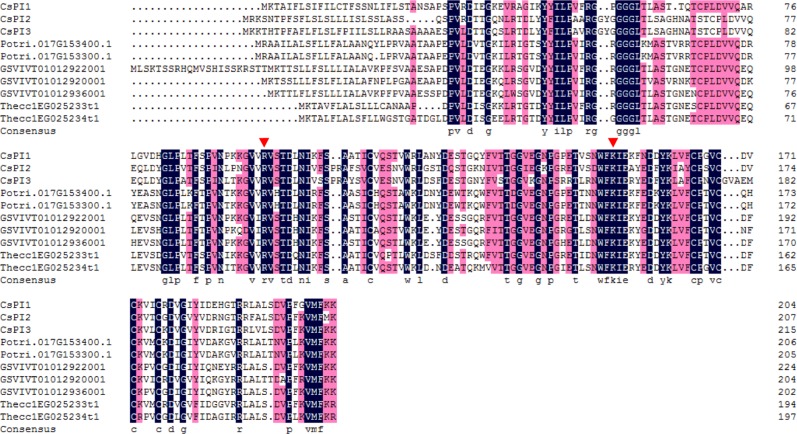
Multiple sequence alignments of three CsKPI proteins with KPI proteins from other plant species. Protein sequences are aligned using DNAMAN software. The consensus amino acid residues are marked with letters at the bottom. Red triangles indicate the two conserved amino acid residues proposed to be essential for disulfide bond formation

To identify tea *CsKPI* genes, KPI amino acid reference sequences from four closely related species were used to search the tea genome (*C. sinensis*)^3^. Three *CsKPIs* were identified and then validated by both reverse transcription polymersae chain reaction (RT-PCR) and Sanger sequencing. The full-length sequence of *CsKPI2* was obtained by rapid amplification of cDNA ends (RACE). To explore important structural regions of CsKPI proteins, we aligned three CsKPIs with seven KPIs from *Theobroma cacao* (2), *Vitis vinifera* (3), and *Populus trichocarpa* (2) plant species. The alignment showed that two residues [arginine (Arg, R) and lysine (Lys, K)] were highly conserved across species (Fig. 1). We further observed a high amino acid sequence similarity (75%) between *CsKPI2* and *CsKPI3*, but *CsKPI1* was less similar to *CsKPI2* (53%) and *CsKPI3* (51%); this suggested that *CsKPI1* may have functionally diverged from *CsKPI2* and *CsKPI3*.

**Fig. 2:**
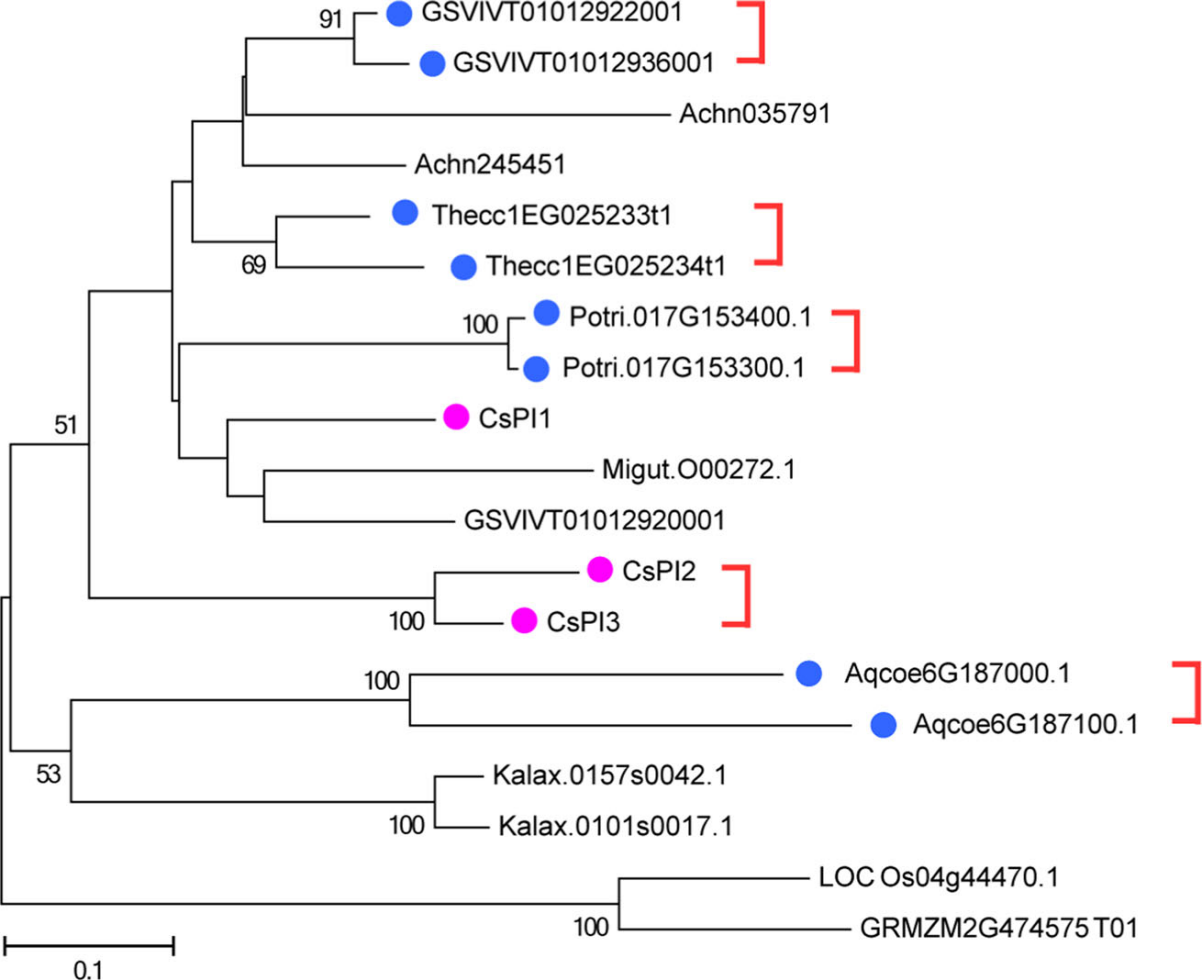
Phylogenetic tree of *CsKPI* genes in tea plant. Nineteen KPI proteins from ten plant species were used to construct an unrooted neighbor joining phylogenetic tree. The bootstrap values of the confidence levels are shown as percentages at branch nodes. The *CsKPI* genes are highlighted in purple. The red half-frame indicates a tandem duplication event between the two framed genes. The solid blue circles indicate tandem duplications of genes from other plant species

To investigate the evolutionary relationship of *CsKPIs* among plant species, we constructed a phylogenetic tree using a total of 19 *KPI* gene sequences from ten plant species (Fig. 2). The 19 *KPIs* were divided into two clusters based on their evolutionary relationship and sequence similarities, and the three *CsKPIs* were grouped into the eudicot category. Moreover, *CsKPI2* and *CsKPI3* were tandemly located within the same scaffold (Supplementary Table. S1). This suggested that a tandem duplication event occurred between these two genes 26.78 MYA, but the separation between these two genes and *CsKPI1* occurred 72.94 MYA (Supplementary Fig. S1). A similar duplication event was observed in some eudicots, such as *P. trichocarpa* (Potri.017G153400.1 and Potri.017G153300.1)*, V. vinifera* (GSVIVT01012922001 and GSVIVT01012936001), *T. cacao* (Thecc1EG025233t1 and Thecc1EG025234t1), and *Aquilegia coerulea* (Aqcoe6G187000.1 and Aqcoe6G187100.1). Notably, no KPIs were identified in lower plants, such as *Dunaliella salina*, *Physcomitrella patens*, and *Selaginella moellendorffi*i, until the occurrence of grass plants, in which a single *KPI* gene was first identified in *Oryza sativa*.

### Activity and subcellular localization of CsKPI1, CsKPI2, and CsKPI3

**Fig. 3:**
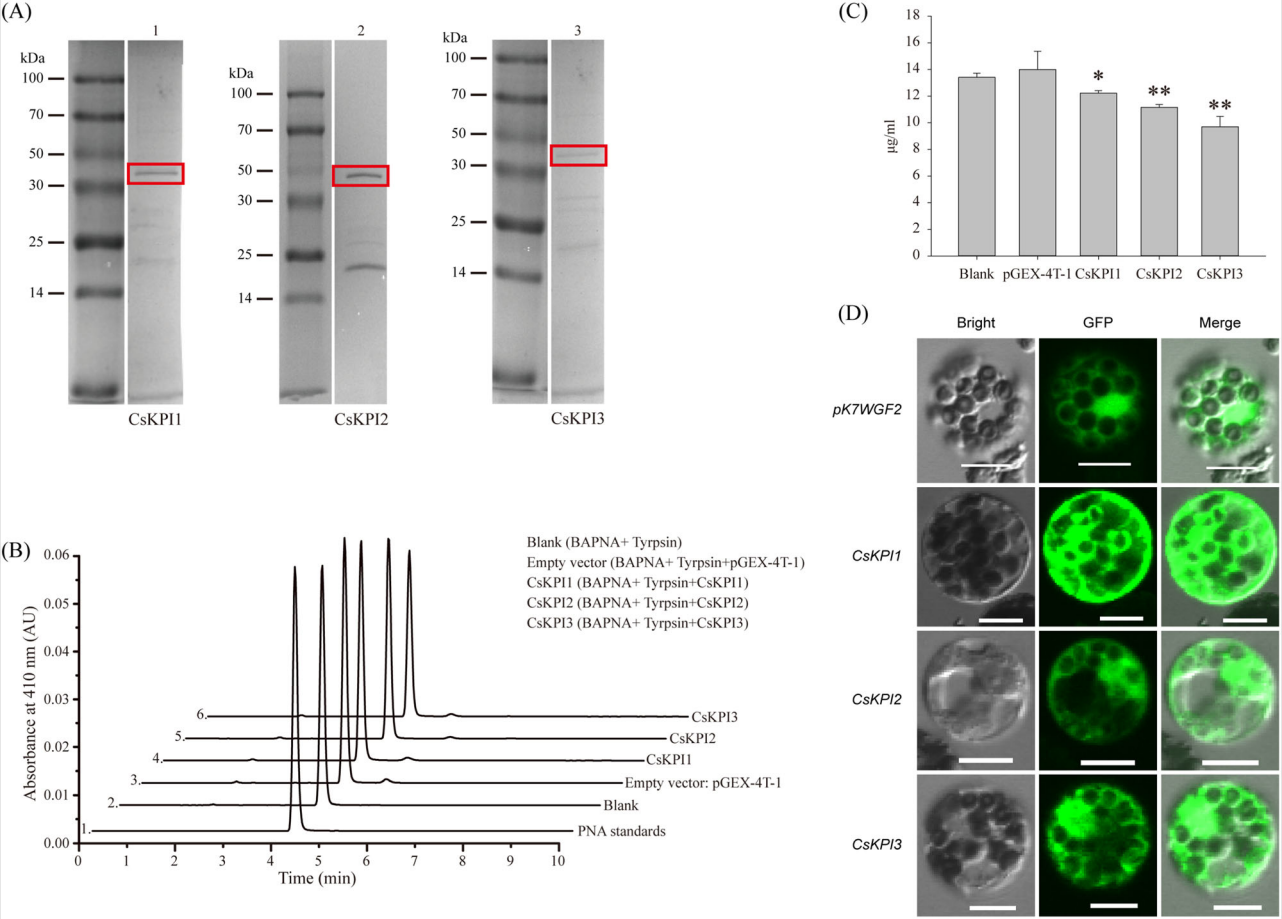
Heterologous expression of *CsKPI* in *E. coli* for determination of enzymatic properties and subcellular localization. **a** SDS-PAGE image of the purified CsKPI1, CsKPI2, and CsKPI3 recombinant proteins: 1, CsKPI1; 2, CsKPI2; and 3, CsKPI3. The solid box shows the expected protein positions. **b** Representative HPLC analyses of enzymatic reaction products after incubating recombinant CsKPI proteins with trypsin and N-α-benzoyl-dl-arginine-4-nitroanilide (BAPNA). Traces 3–6 represent the products from reactions with the recombinant proteins GST, CsKPI1, CsKPI2, and CsKPI3, respectively; traces 1 and 2 represent a standard (PNA) and a blank control group, respectively. **c** Quantitative determination of the PNA content. Bars indicate the means ± SD (*n* = 3) of three biological replicates. Asterisks indicate the significance level (*P *<* 0.05, **P < 0.01) based on Tukey’s honestly significant difference test. **d** Subcellular localization of three CsKPIs. Rows 1–4 show confocal images for the 35:GFP, 35:CsKPI1-GFP, 35:CsKPI2-GFP, and 35:CsKPI3-GFP constructs, which are each transiently expressed in *Arabidopsis* protoplasts. Scale bar = 10 μm

As indicated by the close but distinct phylogenetic relationship between *CsKPI1*, *CsKPI2*, and *CsKPI3*, these genes may exhibit unique enzymatic properties or differ in their biological functions. Heterologous expression and enzymatic measurements were necessary to determine whether the CsKPI1, CsKPI2, and CsKPI3 proteins were functional trypsin inhibitors. We cloned the CDS regions of the three *CsKPIs* into pGEX-4T-1 and obtained the recombinant proteins by heterologous expression of each gene in *E. coli*. Recombinant proteins were purified to improve the efficiency of the enzymatic reactions and to eliminate any interference from nontarget proteins. Sodium dodecyl sulfate polyacrylamide gel electrophoresis analysis indicated that the expressed proteins were of the expected sizes (including the GST tag): CsKPI1, 49 kDa; CsKPI2, 49 kDa; CsKPI3, 50 kDa (Fig. 3a and Supplementary Fig. S2). To investigate the trypsin inhibitory activity of the CsKPI proteins, we performed high-performance liquid chromatography (HPLC) analysis to test the products resulting from mixed enzymatic reactions. Based on their identical reaction conditions, the enzymatic reactions with CsKPI proteins yielded a smaller HPLC peak than those of the blank control sample and the empty vector control sample pGEX-4T-1 (Fig. 3b). Quantitative determination of the PNA content based on peak area and standard curve analysis also supported these results (Fig. 3c), and we concluded that the enzymatic reaction was inhibited by these three CsKPI proteins. Further quantitative determination indicated that the specific inhibitory activities of CsKPI1, CsKPI2, and CsKPI3 recombinant proteins were 499.87, 957.62, and 1694.78 units/g, respectively. To assess the function of the two conserved residues (Arg and Lys), these residues were mutated by site-directed mutagenesis for three CsKPI constructs, and the recombinant proteins were expressed in *E. coli* (Supplementary Fig. S3A). Further results of the enzymatic assay showed that the inhibitory activities of the CsKPI2 and CsKPI3 mutant proteins were significantly decreased relative to those of the respective wild-type proteins; this decrease was not obviously observed in the CsKPI1 mutant protein (Supplementary Fig. S3B, C). To further investigate the subcellular localization of the three CsKPI proteins, we constructed *CsKPI1-GFP*, *CsKPI2-GFP*, *CsKPI3-GFP*, and pK7WGF2 *35S-GFP* fusion protein expression vectors. Transient expression in *Arabidopsis* protoplasts showed that the CsKPI1-GFP, CsKPI2-GFP, and CsKPI3-GFP proteins localized throughout the cytoplasm without distinction (Fig. 3d). Prediction of the signal peptide using SignalP and TargetP software suggested that they may be involved in the secretory pathway (Supplementary Table. S1).

### *CsKPI2* and *CsKPI3* are involved in defense responses against pathogen infection by SA induction

**Fig. 4:**
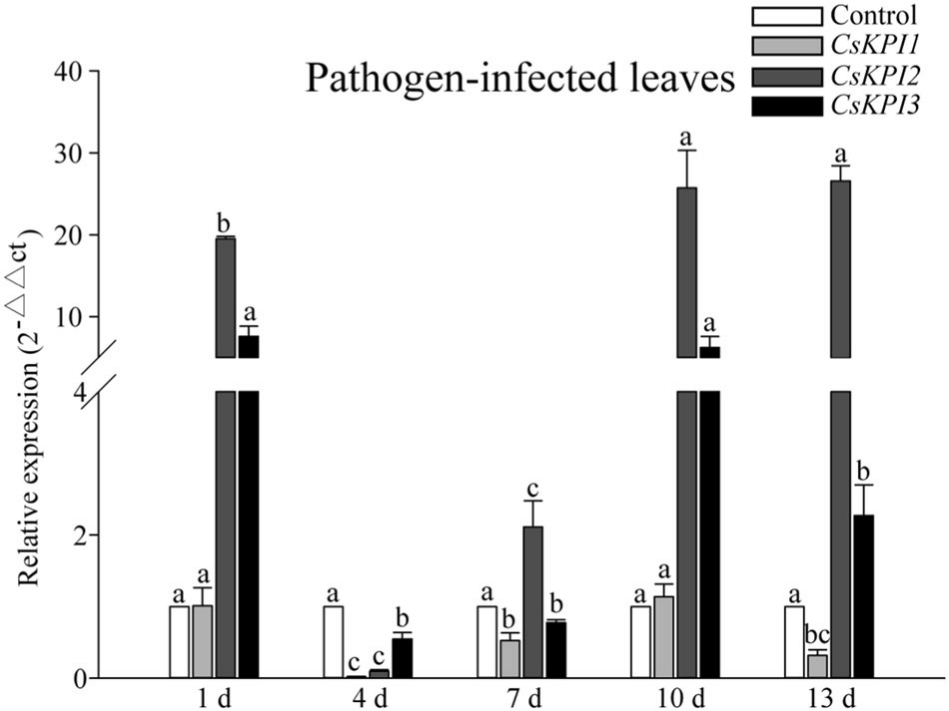
Expression patterns of *CsKPI* genes in leaves infected with *Glomerella cingulata* at different time points. QRT-PCR analysis of *CsKPI* mRNA transcripts in leaves subjected to *Glomerella cingulata* infection. Bars indicate the means ± SD (*n* = 3) of three biological replicates, and bars with different letters are significantly different at *P* < 0.05 according to Duncan’s multiple range test

We explored the expression patterns of *CsKPI* genes in response to *Glomerella cingulata* (fungal causative agent of brown blight disease) infection using qRT-PCR analysis. The three *CsKPIs* were differentially expressed under this treatment. *CsKPI2* and *CsKPI3* were strongly induced at 1 day post inoculation; in particular, the mRNA of *CsKPI2* in infected leaves accumulated up to 19.5-fold relative to that in the untreated control leaves (Fig. 4). Specifically, the expression of *CsKPI2* and *CsKPI3* reached peaks of up to 26.5-fold and 7.6-fold higher than those of the control at 10 days post inoculation, respectively; however, CsKPI1 was only 1.1-fold expression at 10 days post inoculation (Fig. 4), suggesting the functional diversity among them in response to pathogen infection.

**Fig. 5:**
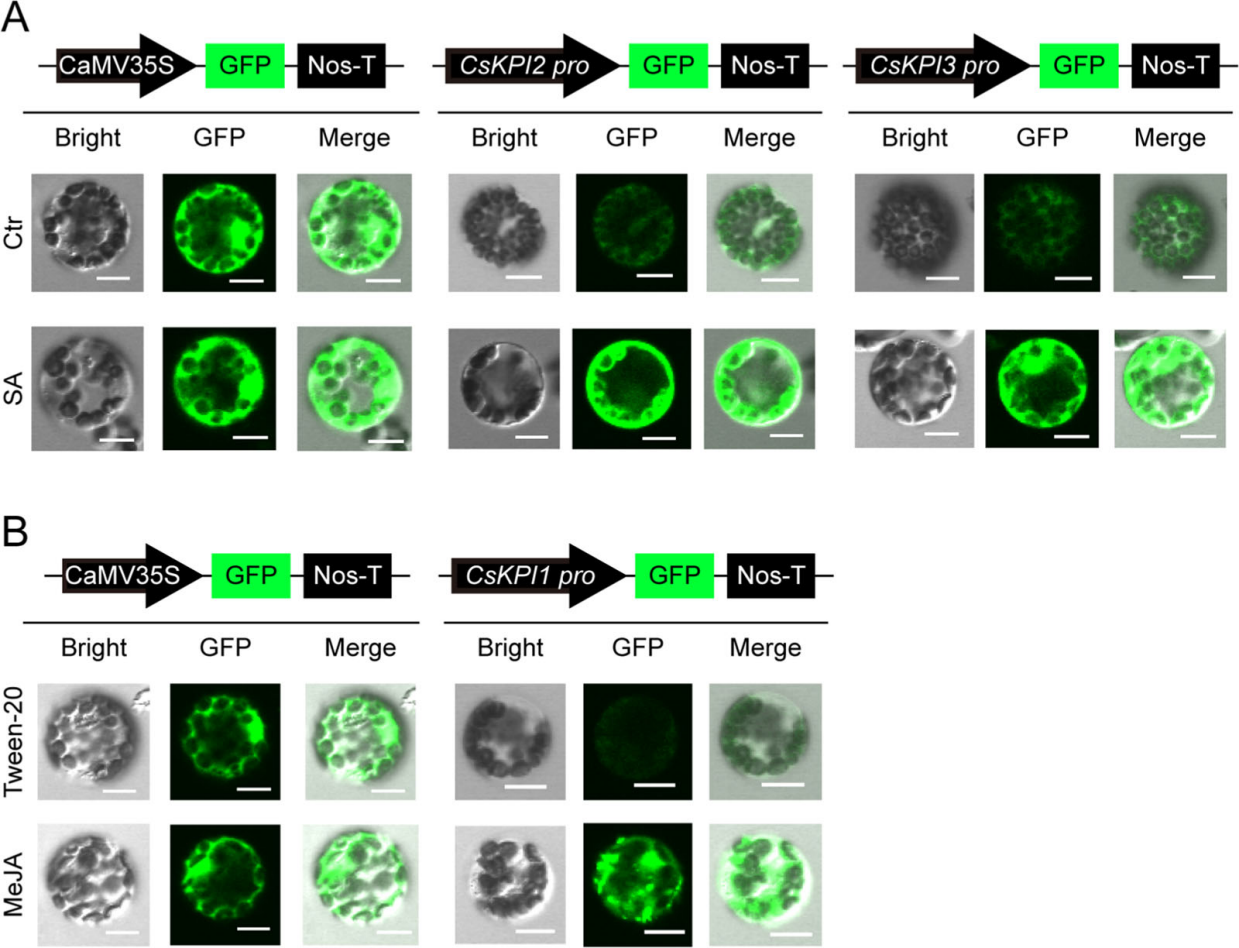
Detection of *CsKPI* promoter activities transiently expressed in *Arabidopsis* protoplasts. GFP reporter constructs containing *CsKPI1*, *CsKPI2*, and *CsKPI3* promoters (*CsKPI1*_*pro*_-GFP, *CsKPI2*_*pro*_-GFP, and *CsKPI3*_*pro*_-GFP) and the CaMV35S promoter (*35S*_*pro*_-GFP; positive control) were used to transiently transfect *Arabidopsis* protoplasts, which were tested for SA (**a**) and MeJA (**b**) induction. After incubation for 8 h, the GFP signal was observed by confocal microscopy. Ctr, control group with sterilized water treatment. Bars = 10 μm

The SA content in infected leaves increased compared to that in healthy leaves (as a control) (Supplementary Fig. S4A), while the ABA, JA, and JA-Ile contents showed nonsignificant increases, respectively (Supplementary Fig. S4B–D). To investigate the influence of SA accumulation on the expression of *CsKPIs*, qRT-PCR was performed. *CsKPI2* and *CsKPI3* were strongly induced at 12 h after SA treatment (Supplementary Fig. S5A); moreover, TCA motifs (SA responsiveness element) were found in the promoter sequences of both of them. To further confirm the induction of *CsKPI2* and *CsKPI3* by SA, the promoters of *CsKPI2* and *CsKPI3* were cloned based on their genomic sequences and placed upstream of a GFP reporter gene and were transiently expressed in *Arabidopsis* protoplast cells with and without SA treatment. The results showed that *CsKPI2*_*p*_*-* and *CsKPI3*_*p*_-driven GFP were strongly expressed in SA-treated protoplast cells but not in untreated cells. In addition, the protoplasts expressing *CaMV35S*_*p*_*::GFP* showed ubiquitous expression in cells irrespective of SA treatment (Fig. 5a).

### JA induced the expression of the *CsKPI1* gene during tea geometrid feeding

**Fig. 6:**
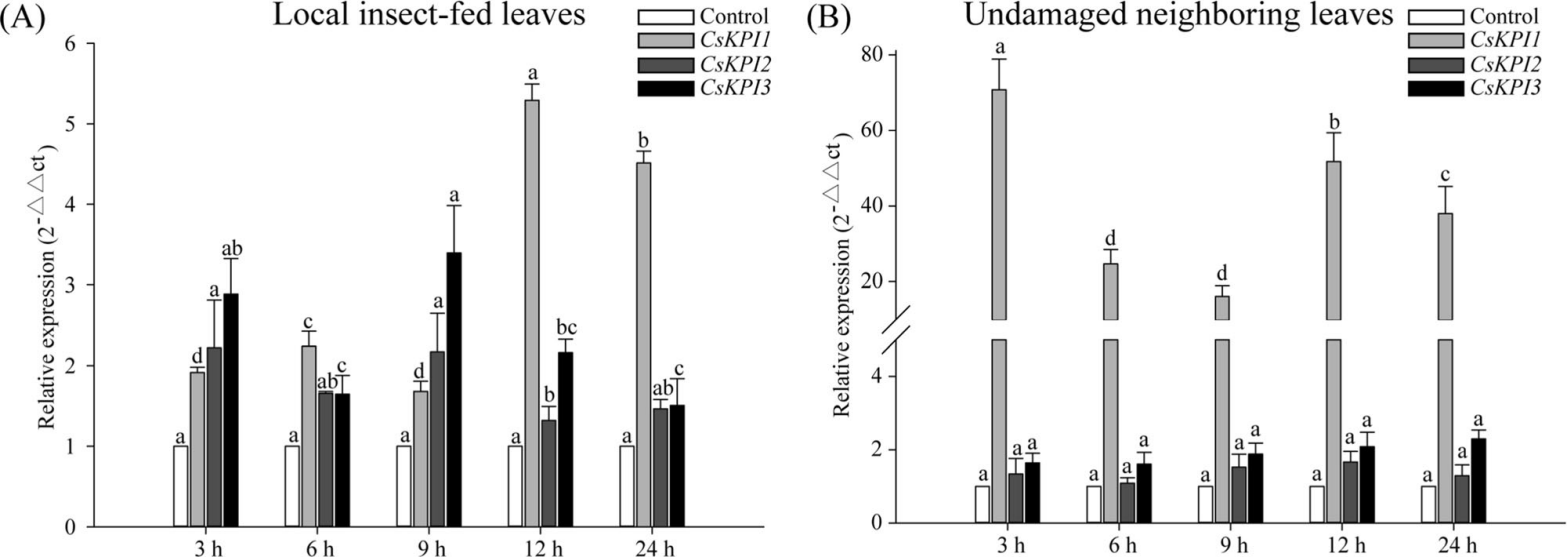
Expression patterns of the *CsKPI* genes under insect feeding treatments at different time points. **a** QRT-PCR analysis of the *CsKPI* genes in damaged leaves under insect feeding treatment. **b** QRT-PCR analysis of the *CsKPI* genes under insect feeding treatment in undamaged neighboring leaves. Bars indicate the means ± SD (*n* = 3) of three biological replicates. Different letters above the bars denote significant differences at *P* < 0.05 according to Duncan’s multiple range test

Since *CsKPI* genes were shown to be involved in insect feeding defense reactions^38^, we used starved tea geometrids to damage healthy leaves. The results of qRT-PCR showed induction of all three *CsKPIs*, but the expression levels of *CsKPI1* quickly (12 h) reached a maximum in localized insect-fed leaves (Fig. 6a). To gain additional insight into the potential signal transduction function of the *CsKPI* genes during insect attack, we also performed qRT-PCR with undamaged neighboring leaves. Notably, *CsKPI1* was not only induced in local insect-fed leaves, but the expression level was substantially higher in undamaged neighboring leaves. For example, the *CsKPI1* mRNA level in insect-fed leaves was up to 1.9-fold that of the control, but a 70.8-fold upregulation was observed in the undamaged neighboring leaves (Fig. 6b). These results suggest that the *CsKPI1* gene may play a role in signal transduction in the tea plant response to tea geometrid attack.

**Fig. 7:**
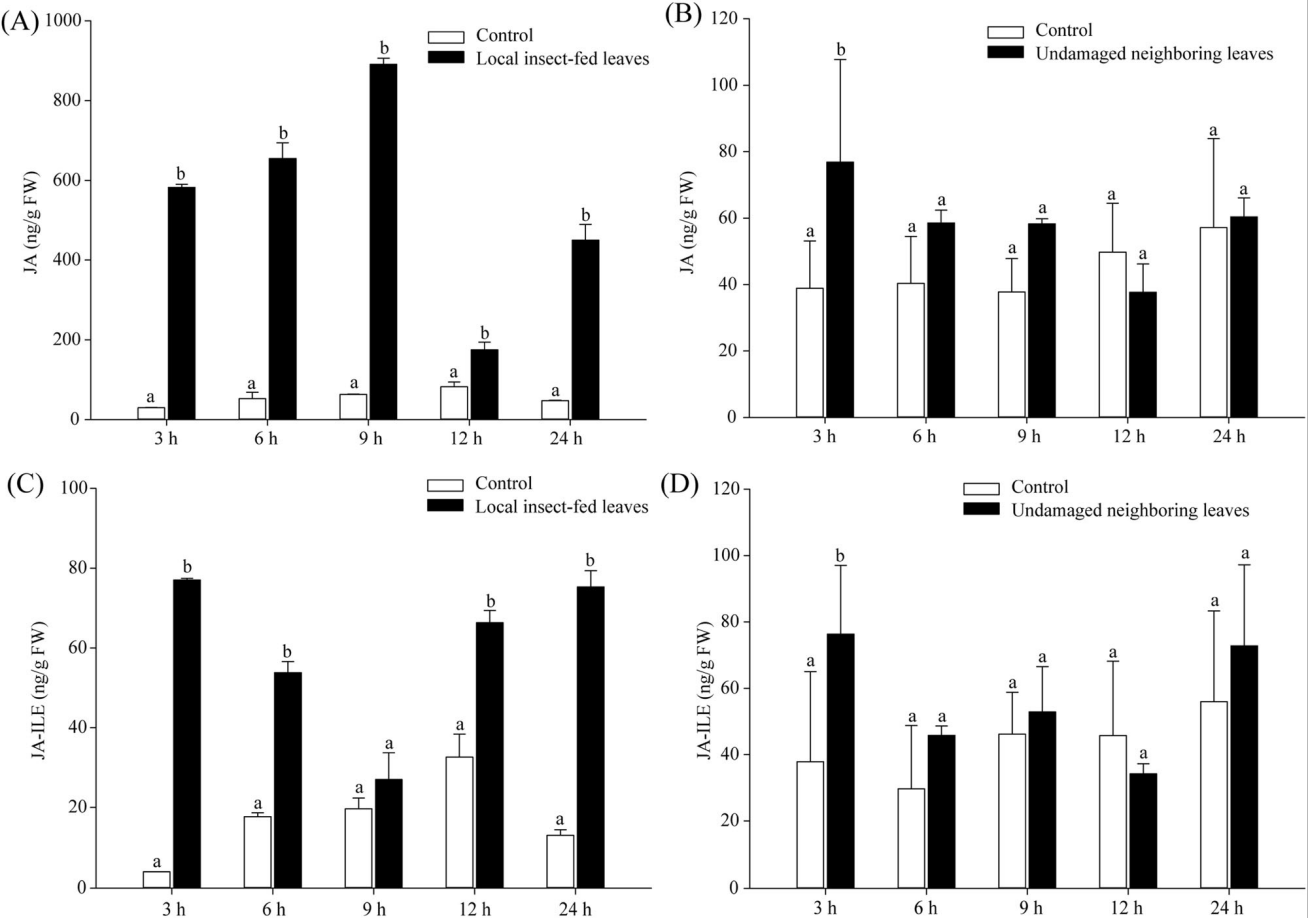
Accumulation of JA and JA-Ile in local insect-fed leaves and neighboring undamaged leaves. Mean JA (**a**, **b**) and JA-Ile (**c**, **d**) concentrations (±SE) in local insect-fed leaves and undamaged neighboring leaves were analyzed with HPLC-MS/MS. Different letters above the bars denote significant differences at *P* < 0.05 according to Duncan’s multiple range test

The accumulation of hormones is vital to signal transduction during plant attack by insects; hence, the contents of phytohormones were measured to investigate whether any of them could induce the expression of *CsKPI1* in tea plant attacked by tea geometrid. We found that the JA and JA-Ile contents were significantly increased in both localized damaged leaves and neighboring undamaged leaves (Fig. 7), but no induction of ABA and SA was observed (Supplementary Fig. S6A–D). Although high contents of JA and JA-Ile were detected in undamaged neighboring leaves, their amounts were higher in localized insect-fed leaves, suggesting an attenuation of the defense signal with time and distance from the source under insect attack. Moreover, *CsKPI1* was upregulated under MeJA treatment in both treated and untreated neighboring leaves, but no induction was observed for *CsKPI2* and *CsKPI3* (Supplementary Fig. S5B, C). This implies that JA acts as a *CsKPI1-*specific signal molecule to modulate the expression of *CsKPI1* and function in SAR defense. In support of these data, MeJA-responsive elements (CGTCA and TGACG motifs) were observed in the *CsKPI1* promoter (Supplementary Table. S2). To determine whether the *CsKPI1* promoter responded to MeJA signals, the CaMV35S promoter was substituted by the *CsKPI1* promoter, and a transient GFP assay was conducted by introducing the chimeric gene into *Arabidopsis* protoplast cells. The GFP signal intensity of the *CsKPI1* promoter in the transfected cells was significantly enhanced at 8 h after treatment, while the signal driven by the CaMV35S promoter was not significantly changed, indicating that *CsKPI1* responded to MeJA signals (Fig. 5b).

## Discussion

PIs have been characterized in many plant species and play key regulatory roles in plant development and in abiotic and biotic stresses^11,33,39^. *KPIs* form a separate subfamily and have been shown to perform vital defensive functions against pests^8^. However, little is known about the transcriptional differentiation of *KPIs* in response to insect attack and pathogen infection in plants. In this study, we identified and cloned three full-length *CsKPI* genes from the tea plant (*C. sinensis*) genome. The inhibitory efficiencies and subcellular localizations of these CsKPI proteins were experimentally verified. We noted differential expression patterns between the three *CsKPIs* in response to tea geometrid feeding and pathogen infection. We concluded that *CsKPI1* was induced during tea leaf feeding by tea geometrids by determining the contents of three hormones in localized insect-damaged and neighboring undamaged leaves and by promoter activity analysis. Moreover, these results implied that pathogen infection activated the expression of *CsKPI2* and *CsKPI3*. Thus, this study provides important details of the divergence of *CsKPIs* in tea plant and represents an example of the evolutionary employment of duplicated genes that seem to have similar physiological functions.

From the absence of *KPIs* in *D. salina* to the presence of three *KPIs* in *C. sinensis*, *KPI* genes have appeared and expanded into a family with the evolution of plants (Fig. 2). Two rounds of WGD occurred in the *C. sinensis* lineage. In contrast, *V. vinifera*, as a species with a close relationship with *C. sinensis*, did not experience a WGD event^3^, and three similar *KPI* genes were found within its genome (Fig. 2). Therefore, the unexpected expansion of the *KPI* gene family in tea plant may not be caused by WGD events. Instead, the appearance of *CsKPI2* and *CsKPI3* were likely caused by a tandem duplication event that occurred 26.78 MYA. These genes have high sequence similarity, share the same location in the genome and have the same expression pattern in response to biotic stresses (Figs. 2, 4, and 5). Similarly, this tandem duplication event was conserved among other closely related plant species, such as *P. trichocarpa* (Potri.017G153400.1 and Potri.017G153300.1), *V. vinifera* (GSVIVT01012922001 and GSVIVT01012936001), and *T. cacao* (Thecc1EG025233t1 and Thecc1EG025234t1) (Fig. 2). The three CsKPI proteins suppress the activity of trypsin and reduce the formation rate of the PNA reaction product (Fig. 3b, c), which mirrors the activities of KPIs from other plant species^40,41^. In addition, the inhibitory activities of the CsKPI2 and CsKPI3 mutant proteins were nearly abolished after two key residues (Arg and Lys) were replaced with alanine, suggesting that these two residues have essential roles in the inhibition of PIs (Supplementary Fig. S3). Notably, these decreases in inhibitory activity were not found in the CsKPI1 protein after replacement of the homologous residues. These large differences in protein properties may result from their low amino acid sequence similarity and the distant evolutionary relationship between CsKPI1 and other CsKPIs. These results also implied that there were undefined key residues in the CsKPI1 protein that influenced its inhibitory activity. Previous studies have indicated that some PI proteins are localized to the plasma membrane, cytoplasm or extracellular space^42–45^. Our transient expression data in *Arabidopsis* protoplast cells showed that all three CsKPI proteins were diffusely distributed in the cytoplasm, nucleus and plasma membrane, rather than localized to one organelle. This may have resulted from the presence of a signal peptide (Fig. 3d and Supplementary Table S1).

In many transgenic plants, *PIs* have been overexpressed to impart resistance against different pathogen infections^46,47^. In this study, *CsKPI2* and *CsKPI3* were strongly induced in response to *G. cingulata* infection (Fig. 4), but no significant transcriptional changes were detected for *CsKPI1*. The results imply that natural selective pressure might result in the transcriptional differentiation between *CsKPI1* and the other two *CsKPIs*, which is similar to the previous observations in the phenylalanine ammonia-lyase and expansin gene families^26,27^. Based on cis-element analysis of *CsKPI2* and *CsKPI3*, the presence of the fungal elicitor element (Box-W1) in the promoter could be a factor for sensitivity to pathogen infection (Supplementary Table S2)^48^. Furthermore, we found that out of the four hormones, only SA accumulated in infected leaves 13 days after inoculation, and SA induced the expression of *CsKPI2* and *CsKPI3* at the transcriptional level (Supplementary Fig. 5A and Fig. 5a). Since SA is a well-known phytohormone regulating disease resistance in plants^29^, our results suggested that SA may be the main hormone signal mediating the expression of *CsKPI2* and *CsKPI3* to defend against pathogen infection. The tandem duplication leading to the *CsKPI2* and *CsKPI3* genes may confer enhanced resistance in tea plant against various pathogens.

In plants, PIs have been commonly considered as anti-insect proteins, which interfere with the digestive processes of insects^38^. Similarly, the expression of the three *CsKPIs* was significantly upregulated during tea geometrid feeding (Fig. 6a), but the highest transcription level was observed for *CsKPI1*. These results further support our hypothesis that transcriptional differentiation occurred after the divergence of the three *CsKPIs* from their common ancestor 72.94 MYA. The recent tandemly duplicated *CsKPI2* and *CsKPI3* genes are primarily involved in defense against pathogen infection, and *CsKPI1* is associated with resistance against insect attack. Furthermore, we simultaneously found that *CsKPI1* was more intensely induced in undamaged neighboring leaves (Fig. 6b). These results suggest that the *CsKPI1* gene not only contributes to the defense response in localized insect-fed leaves but is also involved in SAR during tea geometrid attack.

Studies have shown a linkage between JA accumulation and the expression of *PIs* in response to insect attack^33,49,50^. Moreover, high abundances of JA and *CsKPI1* were simultaneously detected in localized insect-damaged leaves. The contents of JA and JA-Ile both increased in undamaged neighboring leaves (Fig. 7), while no significant accumulation of ABA and SA was detected (Supplementary Fig. S6A–D), suggesting that JA is the vital hormone signal that imparts resistance to herbivory in localized insect-damaged tissue and in the SAR defense response and that JA induces the expression of *CsKPI1* in undamaged neighboring leaves. JA transport may occur within the bulk flow of the phloem or in the transpiration stream, thereby facilitating systemic signal transmission^51,52^. In support of our hypothesis, the JA derivative MeJA could induce the expression of the *CsKPI1* promoter and initiate the expression of a downstream GFP reporter gene (Supplementary Fig. 4B). JA would then mediate a signaling cascade to activate the expression of *CsKPI1* and result in SAR to initialize the various system-wide defense responses in tea plant.

**Fig. 8:**
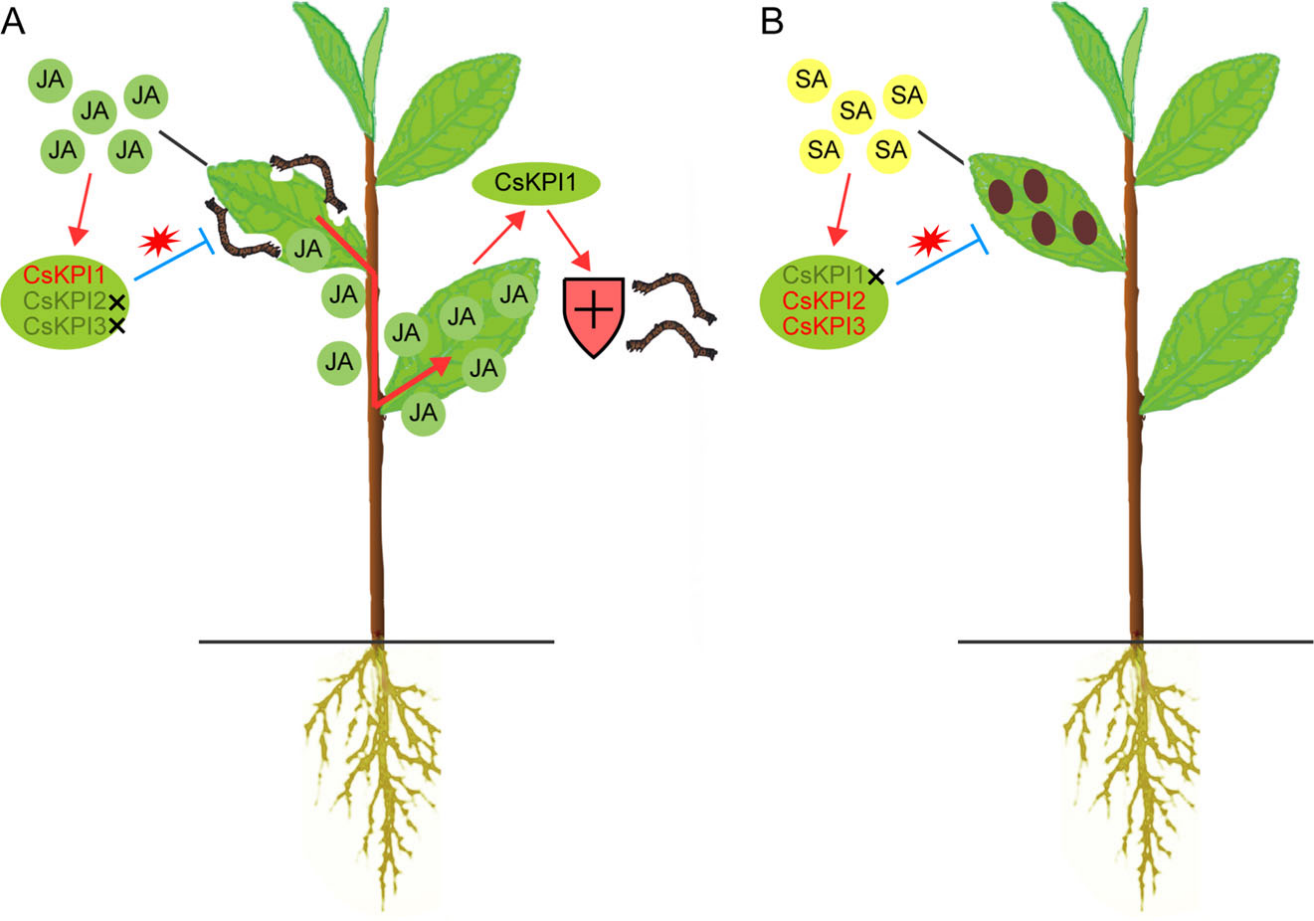
A hypothetical model for function of *CsKPI* genes in tea plant. **a** Tea geometrid feeding stimulates the accumulation of JA, which subsequently induces the expression of *CsKPI1* to defend against tea geometrid attack by repressing their digestive systems. The JA produced in local insect-fed leaves would be transported into undamaged neighboring leaves or biosynthesized de novo to induce *CsKPI1* expression and trigger a broader defense response against tea geometrids. **b** *Glomerella cingulate* induces the accumulation of SA, which activates the transcription of *CsKPI2* and *CsKPI3* to defend the tea leaves from pathogen infection

In conclusion, we identified three *CsKPIs* in tea plant, investigated their evolutionary relationship, and characterized these *CsKPIs* by heterologous expression. *CsKPI2* and *CsKPI3* were generated through tandem duplication and show similar expression patterns that facilitate the tea plant defense response to pathogen infection. In contrast, *CsKPI1* transcriptionally differs from *CsKPI2* and *CsKPI*3 and participates in the SAR response, which requires JA accumulation resulting from tea geometrid feeding (Fig. 8).

## Materials and methods

### Plant materials and treatments

Two-year-old tea plants (*Camellia sinensis* cv. Shuchazao) were obtained from the Dechang tea plantation (Shucheng, latitude 31.3°N, longitude 117.2°E, Anhui, China). Tea plants were grown outdoors in an experimental plot with 120 cm between rows and 33 cm between plants within a row. All tea plants were watered and fertilized with the same routine. The plants chosen for our experiments had uniform heights and canopy widths and were without signs of disease and insects.

Tea geometrids were captured in fields at the Dechang tea plantation and were identified as *Ectropis oblique*. A population of tea geometrids was reared in a growth chamber (temperature: 25 °C, photoperiod: 14/10 h (day/night), humidity: 65 ± 5%). For the insect feeding treatment, 20 tea geometrids at the third larval stage were placed on selected leaves of each individual tea plant. After 1/3 of each leaf was consumed, all tea geometrids were removed, and then the consumed leaves and the undamaged neighboring leaves below them were collected at 3, 6, 9, 12, and 24 h. The samples in the control group without tea geometrid feeding were collected at the same time points (Supplementary Fig. S7A).

The *G. cingulata* fungal strain was obtained from our laboratory’s strain store room. The tea plants were infected according to a previously published protocol developed by our team^53^. Plants were incubated in the greenhouse (temperature: 28 ± 3 °C, photoperiod: 16/8 h (day/night), humidity: 85 ± 5%). The control group consisted of plants with healthy leaves that were inoculated with sterile water. Infected and uninfected control leaves were collected on days 1, 4, 7, 10, and 13 days (Supplementary Fig. S7B).

Leaves of healthy tea plants were wiped with 1 mM MeJA (in 0.05% Tween-20) or 1 mM SA until the leaves were completely wet. Leaves treated with 0.05% Tween-20 and double-distilled water served as the controls for both treatments. The treated leaves and the untreated neighboring leaves below them were collected at 3, 6, 9, 12, and 24 h (Supplementary Fig. S7C).

Three biological replicates were performed for all treatments, and all collected samples were immediately frozen in liquid nitrogen and stored at −80 °C until use.

### Bioinformatics analysis

To investigate the evolutionary divergences among the three *CsKPI*s, synonymous substitution rates (Ks) of three *CsKPI*s were calculated using KaKs_Calculator 2.0 based on the MA model. The divergence time was converted based on the calculated Ks value according to *T* = *Ks*/2*r*, where *r* indicates a substitution rate of 6.5 × 10^−9^ mutations per site per year for eudicots.

MEGA 6.0 software was employed to construct a phylogenetic tree after the multiple sequence alignment analyses of CsKPIs. In the phylogenetic tree, 19 KPI CDS protein sequences were analyzed as candidate genes using the neighbor joining method set to the following parameters: complete deletion, uniform rates, Poisson model, bootstrap method (1000 replicates). The gene accession information for all genes is listed in Supplementary Table S3.

### Total RNA extraction and qRT-PCR analysis

The RNAprep Pure Plant Kit (cat DP441, Tiangen) was used to extract total RNA from plant materials according to the manufacturer’s instructions. The PrimeScript RT Reagent Kit (cat 6110A, Takara) was used to synthesize first-strand cDNA from total RNA. QRT-PCR was performed with a protocol previously reported by our team^6^. All gene-specific primers (P1–P8) are listed in Supplementary Table S4. The amplification efficiencies of all genes used in this study ranged from 95 to 105%^54^. DPS software was used to perform the statistical analysis of qRT-PCR data^55^.

### Gene cloning of full-length *CsKPI* genes and site-directed mutagenesis

Putative *CsKPIs* were obtained based on the current assembly of *C. sinensis* genome sequences^3^. The full-length sequence (P9-P10) was obtained using a SMART RACE Kit (cat 6107, Clontech, Dalian, China) (Supplementary Table S4). Gene-specific primers (P11–P16) were used to amplify full-length *CsKPI* gene sequences (Supplementary Table S4), and the *CsKPI* genes were separately cloned into the pEASY-T1 vector and verified by DNA sequencing.

Site-directed mutagenesis was performed on the *CsKPI* vectors to yield *CsKPI1* (R98A, K152A), *CsKPI2* (R99A, K155A), and *CsKPI3* (R104A, K160A). In brief, three pairs of site-mutant primers were designed and used to amplify three DNA fragments, and then targeted CsKPI fragments were obtained by fusing the three DNA fragments using end-to-end PCR. The PCR products containing mutations were separately cloned into the pEASY-T1 vector and verified by DNA sequencing; the mutagenesis primers are listed in Supplementary Table S4.

### Heterologous expression and purification of the CsKPI proteins

FastPfu polymerase (cat AP221, Transgen, Beijing, China) was used to amplify the ORFs of *CsKPI1*, *CsKPI2, CsKPI3*, and the three corresponding mutated *CsKPIs* using gene-specific primers (P17–P22) (Supplementary Table S4). The pGEX-4T-1 vector was selected as the expression vector; the six target genes (wild types and *CsKPI* mutants) and the vector were digested with the restriction enzymes BamHI (1010A, Takara) and XhoI (1094A, Takara). The ClonExpressII One Step Cloning Kit (cat C112, Vazyme) was used for the assembly according to the manufacturer’s instructions. The constructed plasmids were transformed into *E. coli BL21* chemically competent cells (cat CD601, Transgen). GST resin (cat DP201, Transgen) was used to purify the GST-tagged recombinant proteins by affinity chromatography.

### Enzyme activity assay

The substrate BAPNA (B3133, Sigma Aldrich) was dissolved in dimethylsulfoxide (DMSO) and added to 50 mM Tris-HCL, 25 mM CaCl_2_, pH 8.0, to a final concentration of 0.4 mg/mL. BAPNA (200 µL, 0.4 mg/mL) and the purified protein solution (100 µL, 0.4 µg/µL) were mixed and incubated at 37 °C for 10 min. Trypsin (200 µL, 250 U/µL) was then added, and the mixture was incubated at 37 °C for another 30 min. Finally, 30% glacial acetic acid (100 µL) was added to stop the enzymatic reaction. The catalytic reaction product PNA was measured by HPLC, which was performed on a Waters series HPLC system with a C18 column and ultraviolet detection set at 410 nm. The mobile phase consisted of 1% acetic acid aqueous solution-acetonitrile (50:50 v/v) at a flow rate of 1 mL/min^40,41^. The column oven temperature was set at 25 °C.

### Subcellular localization of the GFP fusion proteins

Gene-specific primers, P23–P28 (Supplementary Table S4), were used to amplify ORFs of *CsKPI1*, *CsKPI2*, and *CsKPI3*, and the PCR products were separately inserted into the pK7WGF2 vector by the BP and LR reactions using Gateway Technology. The resultant plasmids *pK7WGF2-KPI1*, *pK7WGF2-KPI2*, and *pK7WGF2-KPI3* were transformed into *Arabidopsis* protoplast cells according to a previously reported protocol^56^. After overnight transformation, protoplasts were examined using an Olympus FV1000 confocal microscope (Olympus, Japan).

### Analysis of phytohormone concentrations

The JA, JA-Ile, ABA, and SA concentrations were measured in three biological replicates according to reported studies^57^. Approximately, 150 mg of frozen leaf material was ground in liquid nitrogen; ethyl acetate (1 mL) containing D_5_-JA, ^13^C_6_-JA-Ile, D_6_-ABA, and D_4_-SA was added to each sample to serve as internal standards. Samples were mixed and then centrifuged at 13,000*g* for 10 min at 4 °C. The supernatants were evaporated to dryness in a vacuum concentrator (Eppendorf, Germany). The residues from the samples were resuspended in 0.4 mL of 70% methanol (v/v), and the samples were centrifuged again to clarify the phases. Phytohormone concentrations were measured on an LCMS-8040 (Shimadzu, Japan) with a Shim-pack XR-ODS column (2.0 × 75 mm, 2.2 μm) (Shimadzu, Japan). The column temperature was set at 40 °C, and the flow rate was 0.27 mL/min.

### Promoter activity assays

The CP Plant Miniprep Kit (cat GD2621-01, BIOMIGA) was used to extract genomic DNA from tea leaves according to the manufacturer’s instructions. The promoters of *CsKPI1*, *CsKPI2*, and *CsKPI3* were amplified from genomic DNA with FastPfu polymerase using promoter-specific primers (P29–P34) (Supplementary Table S4). The *CsKPI1*, *CsKPI2*, and *CsKPI3* promoter amplicons were separately inserted into the *pCAMBIA1302-GFP* vector by replacing the *CaMV35S* promoter, and they were then used to transform *Arabidopsis* protoplasts as previously described^58^. Transformed protoplasts were treated with 0.05% Tween-20 (control), 50 μM MeJA and 50 μM SA for 8 h at 25 °C. The GFP signal was detected after phytohormone treatment using an Olympus FV1000 confocal microscope.

## Supplementary information

Supplementary
