# Supplementary material for: Duplication and transcriptional divergence of three Kunitz protease inhibitor genes that modulate insect and pathogen defenses in tea plant (Camellia sinensis)
Source: Hortic Res. 2019 Nov 15;6:126. doi: 10.1038/s41438-019-0208-5 (PMC6856355; doi:10.1038/s41438-019-0208-5)
Supplement: Supplementary file 1 — Supplementary [file 41438_2019_208_MOESM1_ESM.doc]

**Supplementary data**


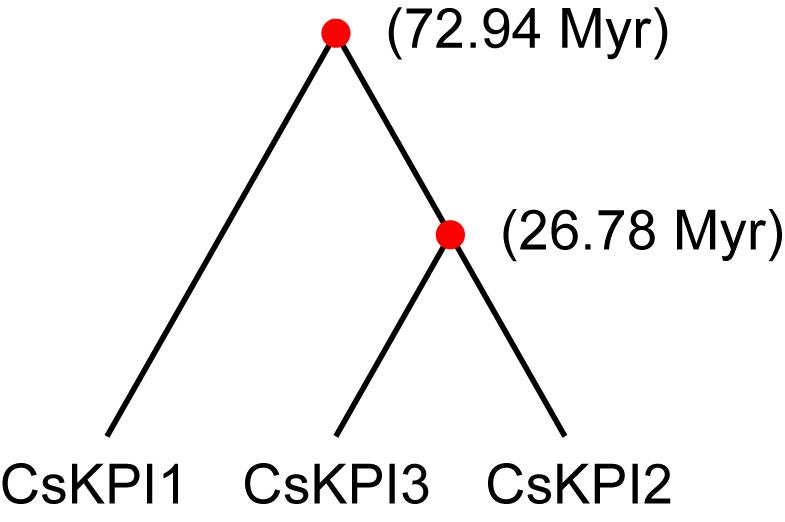


**Supplementary Fig. S1** Evolution of *KPI* genes in tea plant (*Camellia sinensis*). The numbers in brackets indicate the estimated duplication dates for divergence events.


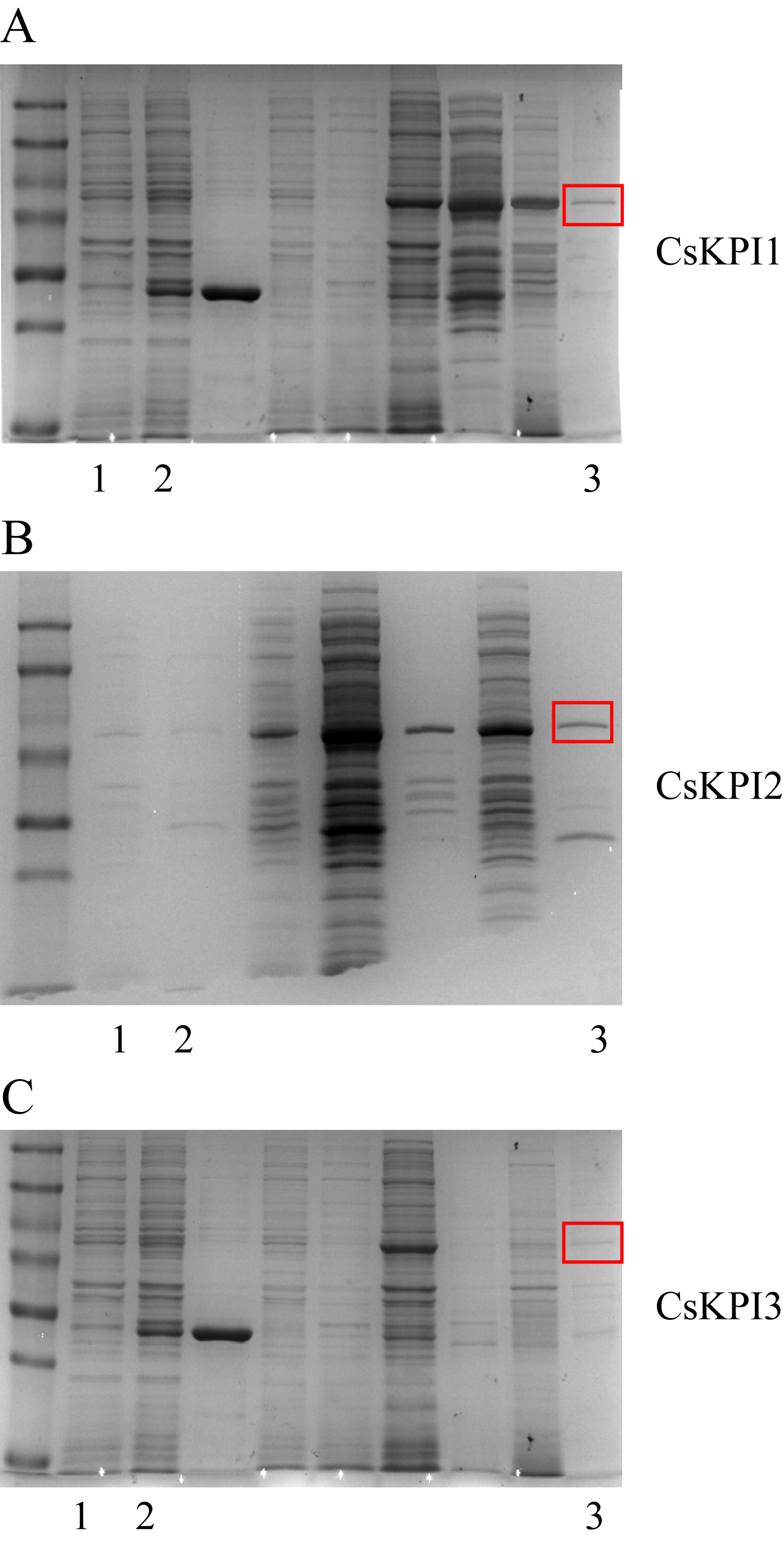


**Supplementary Fig. S2** Heterologous expression of CsKPI in E. coli. (A) SDS-PAGE image of purified CsKPI1 recombinant protein. 1, protein extracted from empty pGEX-4T-1 before induction. 2, protein extracted from empty pGEX-4T-1 after induction. 3, purified CsKPI1. (B) SDS-PAGE image of purified CsKPI2 recombinant protein. 1, protein extracted from empty pGEX-4T-1 before induction. 2, protein extracted from empty pGEX-4T-1 after induction. 3, purified CsKPI2. (C) SDS-PAGE image of purified CsKPI3 recombinant protein. 1, protein extracted from empty pGEX-4T-1 before induction. 2, protein extracted from empty pGEX-4T-1 after induction. 3, purified CsKPI3. The solid box shows the position of expected protein.


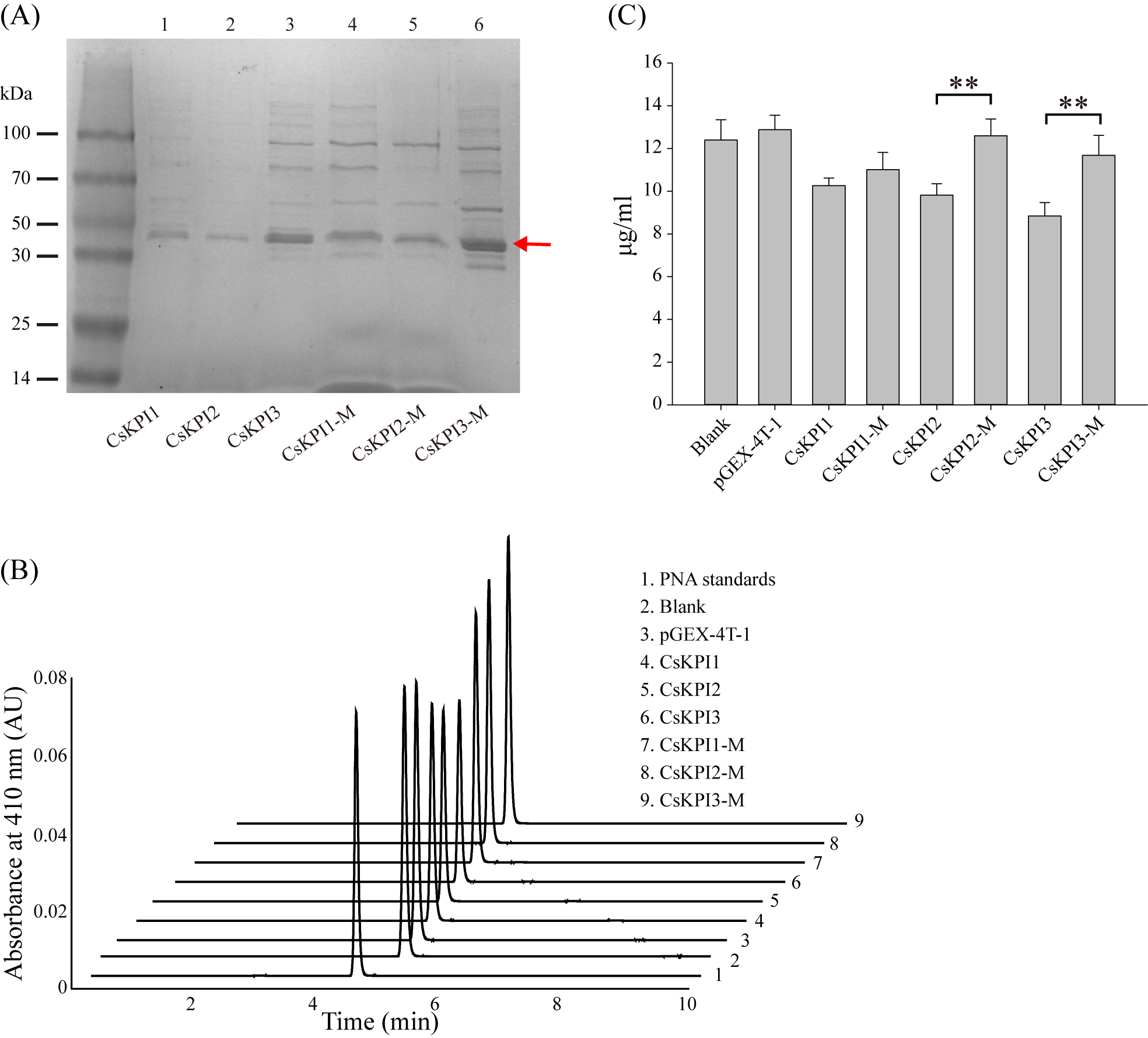


**Supplementary Fig. S3** Heterologous expression of *CsKPI* in *E. coli* for determination of enzymatic properties. (A) SDS-PAGE image of the purified recombinant proteins CsKPI1, CsKPI2, CsKPI3 (wt and with site specific mutations to conserved residues): 1, full-length CsKPI1; 2, full-length CsKPI2; 3, full-length CsKPI3; 4, CsKPI1 (R98A, K152A); 5, CsKPI2 (R99A, K155A); 6, CsKPI3 (R104A, K160A). Expected protein positions indicated by red arrow. (B) Representative HPLC analysis of enzymatic reaction products after incubating recombinant CsKPI proteins with trypsin and N-α-benzoyl-DL-arginine-4-nitroanilide (BAPNA). Traces 3, 4, 5, 6, 7, 8 and 9 represent the PNA product from reactions containing BAPNA, trypsin and recombinant proteins GST, full-length CsKPI1, full-length CsKPI2, full-length CsKPI3, CsKPI1 (R98A, K152A);, CsKPI2 (R99A, K155A) and CsKPI3 (R104A, K160A), respectively; traces 1 and 2 represent a standard (PNA) and a blank control group, respectively. (C) Quantitative determination of PNA content. Bars indicate the means±SD (n= 3) of three biological replicates. Asterisks indicate the significance level (**P* < 0.05, ***P* < 0.01) based on a Tukey’s honestly significant difference test.


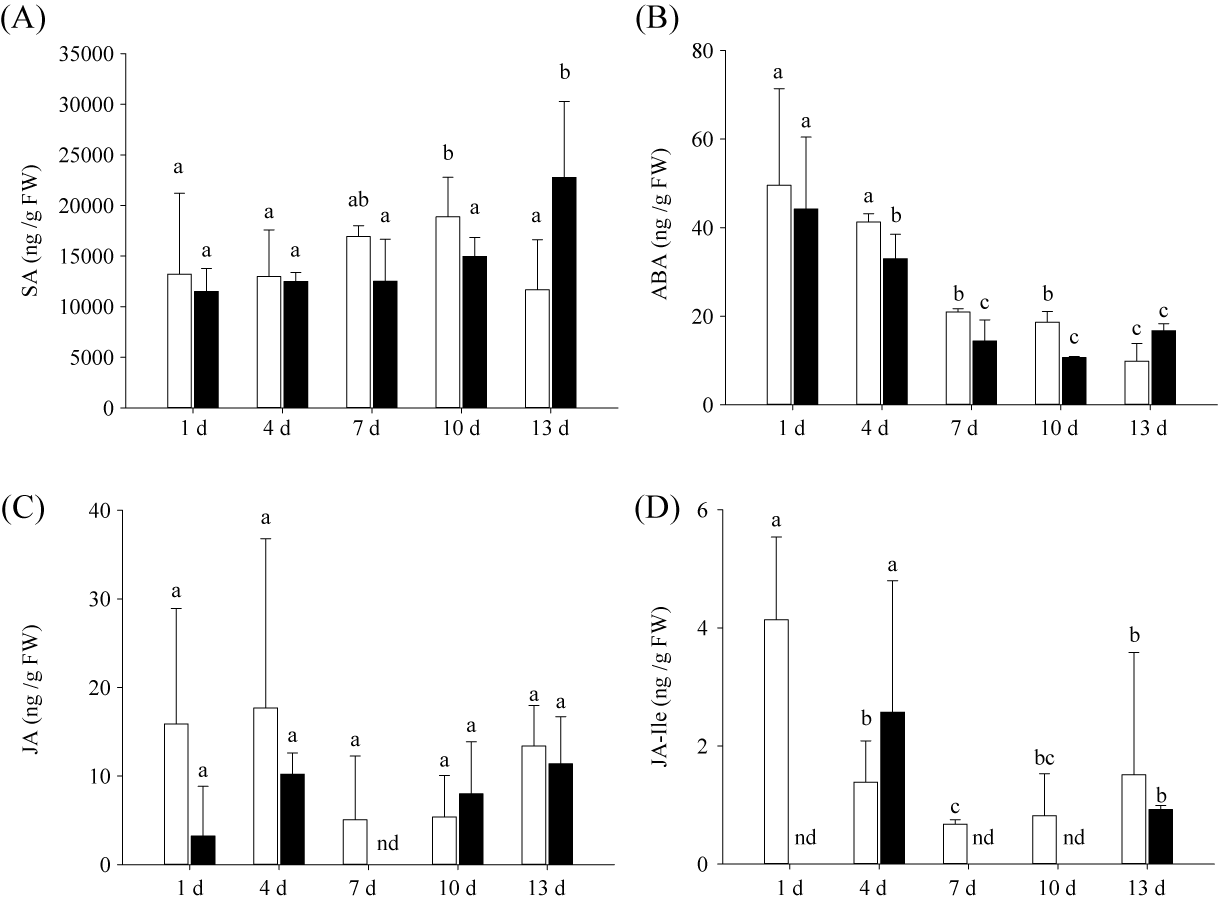


**Supplementary Fig. S4** Accumulation of phytohormones in pathogen infected leaves. Mean SA (A), ABA (B), JA (C) and JA-Ile (D) concentrations in pathogen infected leaves were analyzed by HPLC-MS/MS. Bars indicate the means±SD (n= 3) of three biological replicates. Different letters above the bars denote significant differences at *P* < 0.05 according to Duncan's multiple range test.


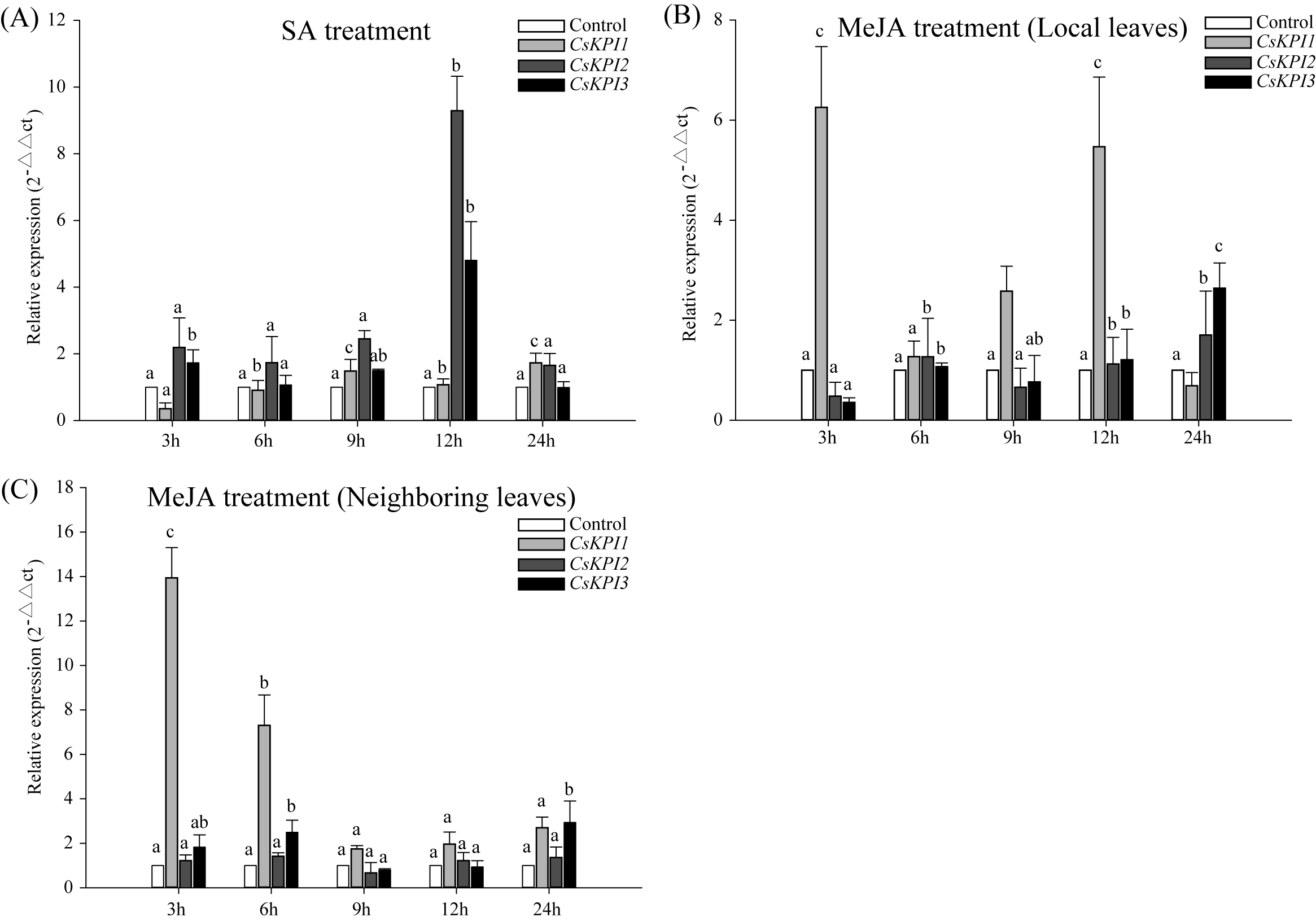


**Supplementary Fig. S5** Expression patterns of *CsKPI* genes under phytohormone treatments at different time points. (A) QRT-PCR analysis of *CsKPI* genes under SA treatment in local insect-fed leaves. (B) QRT-PCR analysis of *CsKPI* genes under MeJA phytohormone treatments in leaves. (C) QRT-PCR analysis of *CsKPI* genes under MeJA phytohormone treatments in neighboring leaves. Bars indicate the means±SD (n= 3) of three biological replicates. Different letters above the bars denote significant differences at *P* < 0.05 according to Duncan's multiple range test.


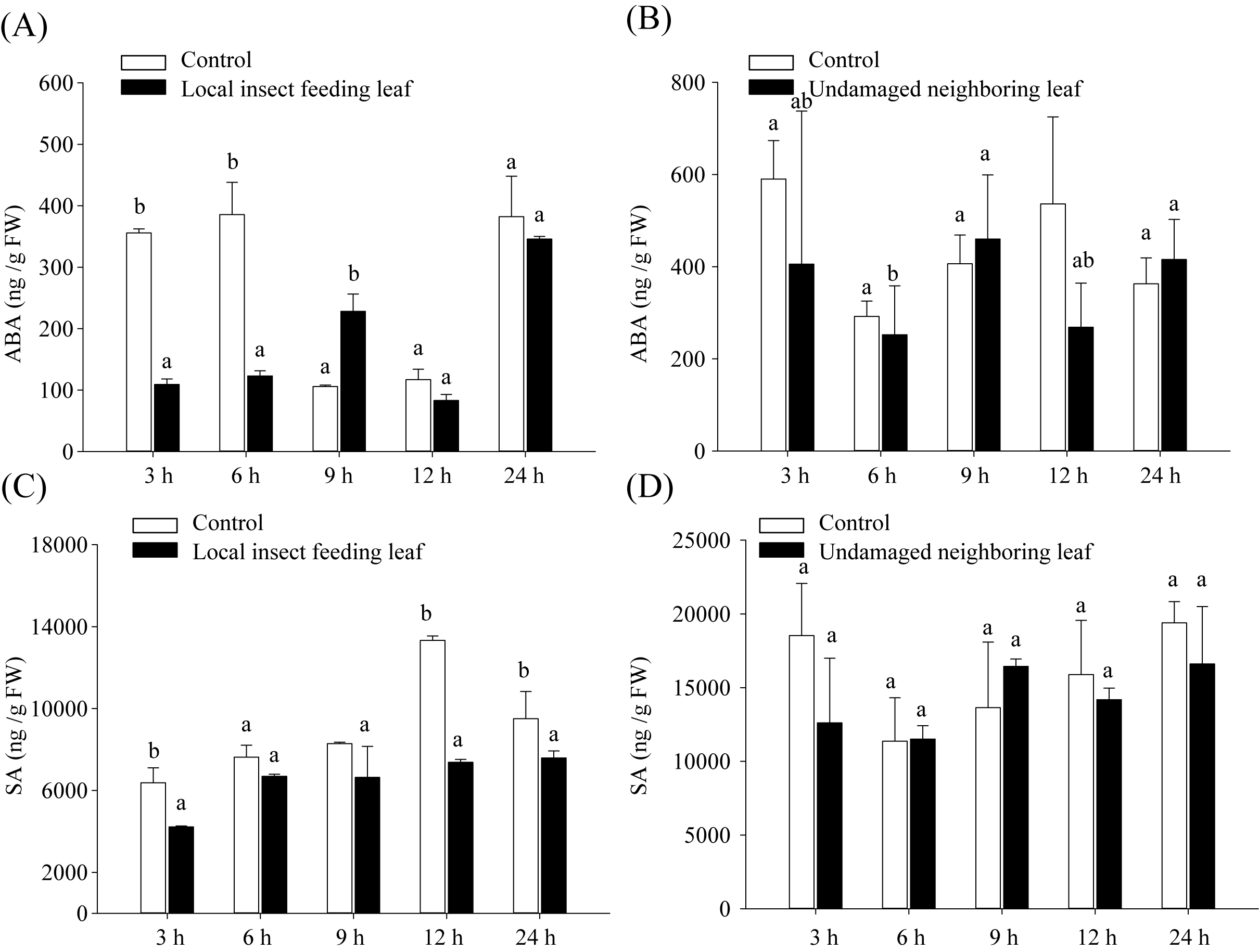


**Supplementary Fig. S6** Accumulation of phytohormones in local insect-fed and undamaged neighboring leaves. Mean ABA (A, B) and SA (C, D) concentrations local insect-fed and undamaged neighboring leaves were analyzed by HPLC-MS/MS. Bars indicate the means±SD (n= 3) of three biological replicates. Different letters above the bars denote significant differences at *P* < 0.05 according to Duncan's multiple range test.


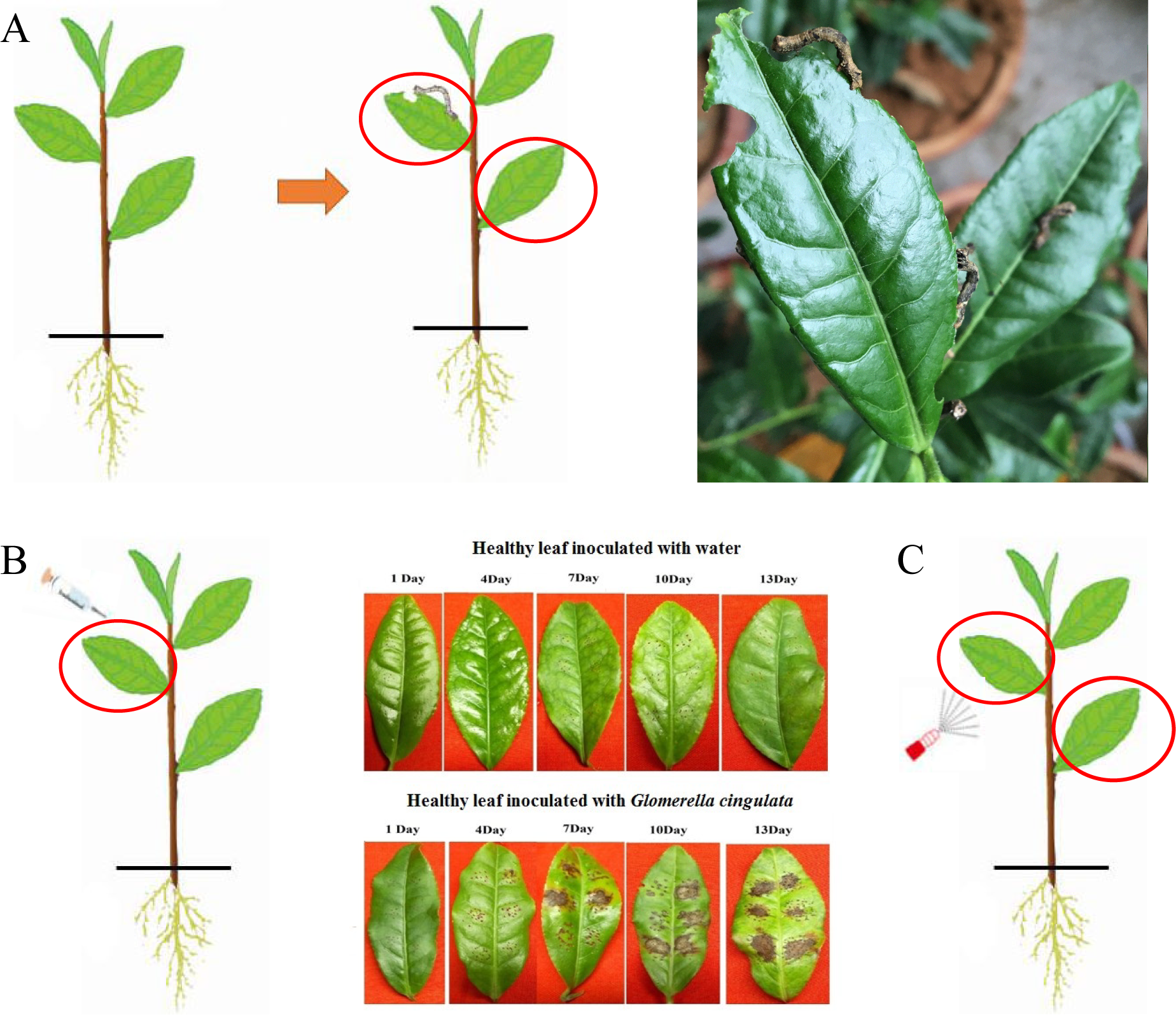


**Supplementary Fig. S7** Schematic diagram of sample collection. (A) The tea plant was fed upon by tea geometrids. (B) The tea plant was infected by Glomerella cingulata strain. (C) Tea plants were treated with MeJA and SA, respectively. Red circles indicate sampling positions on the tea plant. Detailed information of sampling is described in materials and methods.

| ID | CDS length (bp) | Predicted Scaffold | Protein | | | Sub-cellular location | |  |
| --- | --- | --- | --- | --- | --- | --- | --- | --- |
|  |  |  | Protein length (aa) | Mol.Wt (kDa) | PI | TargetP | Cell-PLoc | SignalP (signal peptide?) |
| *CsKPI1* | 618 | Scaffold3462 | 205 | 22 | 8.45 | Secretory pathway | Vacuole | Yes |
| *CsKPI2* | 642 | Scaffold628 | 213 | 23 | 8.14 | Secretory pathway | Vacuole | Yes |
| *CsKPI3* | 651 | Scaffold628 | 216 | 23 | 9.02 | Secretory pathway | Vacuole | Yes |

**Supplementary** **Table. S1** Detailed information of three *CsKPI* genes identified in the tea plant genome. Basic characteristic data about CsKPI proteins were calculated using ProtParam tool (http://web.expasy.org/*protparam/).* Sub-cellular localization was predicted through the following online website: TargetP 1.1 (http://www.cbs.dtu.dk/services/TargetP/), and MultiLoc2 (http://abi.inf.uni-tuebingen.de/Services/MultiLoc2).

| Function (CsKPI1) | cis-regulatory element |
| --- | --- |
| light responsiveness | AAAC-motif, ACE, Box I, I-box,G-Box,MRE,chs-CMA2b |
| abscisic acid responsiveness | [ABRE](http://bioinformatics.psb.ugent.be/webtools/plantcare/cgi-bin/show_site_info.htpl?QWhere=ID_of_Site like 'AT~ABRE'&StartAt=0&NbRecs=10) |
| fungal elicitor responsive element | [Box-W1](http://bioinformatics.psb.ugent.be/webtools/plantcare/cgi-bin/show_site_info.htpl?QWhere=ID_of_Site like 'PC~Box-W1'&StartAt=0&NbRecs=10) |
| MeJA-responsiveness | [CGTCA-motif,TGACG-motif](http://bioinformatics.psb.ugent.be/webtools/plantcare/cgi-bin/show_site_info.htpl?QWhere=ID_of_Site like 'HV~CGTCA-motif'&StartAt=0&NbRecs=10) |
| gibberellin-responsive element | [GARE-motif](http://bioinformatics.psb.ugent.be/webtools/plantcare/cgi-bin/show_site_info.htpl?QWhere=ID_of_Site like 'BO~GARE-motif'&StartAt=0&NbRecs=10) |
| heat stress responsiveness | [HSE](http://bioinformatics.psb.ugent.be/webtools/plantcare/cgi-bin/show_site_info.htpl?QWhere=ID_of_Site like 'BO~HSE'&StartAt=0&NbRecs=10) |
| salicylic acid responsiveness | [TCA-element](http://bioinformatics.psb.ugent.be/webtools/plantcare/cgi-bin/show_site_info.htpl?QWhere=ID_of_Site like 'BO~TCA-element'&StartAt=0&NbRecs=10) |
| auxin-responsive element | [TGA-element](http://bioinformatics.psb.ugent.be/webtools/plantcare/cgi-bin/show_site_info.htpl?QWhere=ID_of_Site like 'BO~TGA-element'&StartAt=0&NbRecs=10) |
| ethylene-responsive element |  |
| defense and stress responsiveness |  |
| wound-responsive element | [WUN-motif](http://bioinformatics.psb.ugent.be/webtools/plantcare/cgi-bin/show_site_info.htpl?QWhere=ID_of_Site like 'BO~WUN-motif'&StartAt=0&NbRecs=10) |

| Function (CsKPI2) | cis-regulatory element |
| --- | --- |
| light responsiveness | [AT1-motif,ATCT-motif,TGG-motif,Box 4,Box I,G-Box,GAG-motif,GATA-motif,GT1-motif,Sp1](http://bioinformatics.psb.ugent.be/webtools/plantcare/cgi-bin/show_site_info.htpl?QWhere=ID_of_Site like 'ST~AT1-motif'&StartAt=0&NbRecs=10) |
| abscisic acid responsiveness | [ABRE](http://bioinformatics.psb.ugent.be/webtools/plantcare/cgi-bin/show_site_info.htpl?QWhere=ID_of_Site like 'AT~ABRE'&StartAt=0&NbRecs=10) |
| fungal elicitor responsive element | [Box-W1](http://bioinformatics.psb.ugent.be/webtools/plantcare/cgi-bin/show_site_info.htpl?QWhere=ID_of_Site like 'PC~Box-W1'&StartAt=0&NbRecs=10) |
| MeJA-responsiveness |  |
| gibberellin-responsive element |  |
| heat stress responsiveness | [HSE](http://bioinformatics.psb.ugent.be/webtools/plantcare/cgi-bin/show_site_info.htpl?QWhere=ID_of_Site like 'BO~HSE'&StartAt=0&NbRecs=10) |
| salicylic acid responsiveness | [TCA-element](http://bioinformatics.psb.ugent.be/webtools/plantcare/cgi-bin/show_site_info.htpl?QWhere=ID_of_Site like 'BO~TCA-element'&StartAt=0&NbRecs=10) |
| auxin-responsive element |  |
| ethylene-responsive element | ERE |
| defense and stress responsiveness | [TC-rich repeats](http://bioinformatics.psb.ugent.be/webtools/plantcare/cgi-bin/show_site_info.htpl?QWhere=ID_of_Site like 'NT~TC-rich repeats'&StartAt=0&NbRecs=10) |
| wound-responsive element |  |

| Function (CsKPI3) | cis-regulatory element |
| --- | --- |
| light responsiveness | [ACE,AE-box,AT1-motif,Box 4,GA-motif,GT1-motif,L-box](http://bioinformatics.psb.ugent.be/webtools/plantcare/cgi-bin/show_site_info.htpl?QWhere=ID_of_Site like 'PC~ACE'&StartAt=0&NbRecs=10) |
| abscisic acid responsiveness |  |
| fungal elicitor responsive element | [Box-W1](http://bioinformatics.psb.ugent.be/webtools/plantcare/cgi-bin/show_site_info.htpl?QWhere=ID_of_Site like 'PC~Box-W1'&StartAt=0&NbRecs=10) |
| MeJA-responsiveness |  |
| gibberellin-responsive element | [P-box](http://bioinformatics.psb.ugent.be/webtools/plantcare/cgi-bin/show_site_info.htpl?QWhere=ID_of_Site like 'OS~P-box'&StartAt=0&NbRecs=10) |
| heat stress responsiveness | [HSE](http://bioinformatics.psb.ugent.be/webtools/plantcare/cgi-bin/show_site_info.htpl?QWhere=ID_of_Site like 'BO~HSE'&StartAt=0&NbRecs=10) |
| salicylic acid responsiveness | [TCA-element](http://bioinformatics.psb.ugent.be/webtools/plantcare/cgi-bin/show_site_info.htpl?QWhere=ID_of_Site like 'BO~TCA-element'&StartAt=0&NbRecs=10) |
| auxin-responsive element |  |
| ethylene-responsive element |  |
| defense and stress responsiveness | [TC-rich repeats](http://bioinformatics.psb.ugent.be/webtools/plantcare/cgi-bin/show_site_info.htpl?QWhere=ID_of_Site like 'NT~TC-rich repeats'&StartAt=0&NbRecs=10) |
| wound-responsive element |  |

**Supplementary Table. S2** Analysis of cis-acting regulatory elements in the promoter region of CsKPI genes. Cis-acting regulatory elements were annotated using online tool (http://bioinformatics.psb.ugent.be/webtools/plantcare/html/).

| Organism | Sequence ID | GeneBank Accession |
| --- | --- | --- |
| *Oryza sativa* | *LOC_Os04g44470.1* | LOC_Os04g44470.1 |
| *Zea mays* | *GRMZM2G474575_T01* | GRMZM2G474575_T01 |
| *Aquilegia coerulea* | *Aqcoe6G187000.1* | Aqcoe6G187000.1 |
|  | *Aqcoe6G187100.1* | Aqcoe6G187100.1 |
| *Mimulus guttatus* | *Migut.O00272.1* | Migut.O00272.1 |
| *Kalanchoe laxiflora* | *Kalax.0157s0042.1* | Kalax.0157s0042.1 |
|  | *Kalax.0101s0017.1* | Kalax.0101s0017.1 |
| *Actinidia chinensis Planch* | *Achn245451* | Achn245451 |
|  | *Achn035791* | Achn035791 |
| *Populus trichocarpa* | *Potri.017G153400.1* | Potri.017G153400.1 |
|  | *Potri.017G153300.1* | Potri.017G153300.1 |
| *Vitis vinifera* | *GSVIVT01012922001* | GSVIVT01012922001 |
|  | *GSVIVT01012920001* | GSVIVT01012920001 |
|  | *GSVIVT01012936001* | GSVIVT01012936001 |
| *Theobroma cacao* | *Thecc1EG025233t1* | Thecc1EG025233t1 |
|  | *Thecc1EG025234t1* | Thecc1EG025234t1 |
| *Camellia sinensis* | *CsKPI1* | MK057519 |
|  | *CsKPI2* | MK057520 |
|  | *CsKPI3* | MK057521 |

**Supplementary Table. S3** Plant Kunitz type protease inhibitor sequences used for phylogenetic tree construction.

| Primer name | Sequence (5'-3') | Target gene | Purpose |
| --- | --- | --- | --- |
| P1-F | ttggcatcgttgagggtct | *CsGAPDH* | qRT-PCR |
| P2-R | cagtgggaacacggaaagc |
| P3-F | ctctgctcccagcccag | *CsKPI1* | qRT-PCR |
| P4-R | gttgatacccgaaccacgc |
| P5-F | agaaacggtgagcgattgg | *CsKPI2* | qRT-PCR |
| P6-R | catcactgagggcaaacc |
| P7-F | aatcgactggaaagtacttc | *CsKPI3* | qRT-PCR |
| P8-R | aatccgatcaacataaaccc |
| P9-F | caacctcccaaacggcgtagttc | *CsKPI2* | RACE |
| P10-F | aagggaagcctgggagagaaacgg |
| P11-F | atgaagacagcaatattcctctc | *CsKPI1* | CDS amplification |
| P12-R | ttaagccttcttaaacatgactcc |
| P13-F | atgaggaaatcaaacacccccttc | *CsKPI*2 | CDS amplification |
| P14-R | ttaaaccttcatgaacatgactttgaagg |
| P15-F | atgaagaaaacacacactccctt | *CsKPI*3 | CDS amplification |
| P16-R | tcagaccttcttaaacatgactttg |
| P17-F | atctggttccgcgtggatccatgaagacagcaatattcctctc | *CsKPI1* | Heterologous expression |
| P18-R | tcacgatgcggccgctcgagttaagccttcttaaacatgactcc |
| P19-F | atctggttccgcgtggatccatgaggaaatcaaacacccccttc | *CsKPI2* | Heterologous expression |
| P20-R | tcacgatgcggccgctcgagttaaaccttcatgaacatgactttgaagg |
| P21-F | atctggttccgcgtggatccatgaagaaaacacacactccctt | *CsKPI*3 | Heterologous expression |
| P22-R | tcacgatgcggccgctcgagtcagaccttcttaaacatgactttg |
| P23-F | ggggacaagtttgtacaaaaaagcaggcttcatgaagacagcaatattcct | *CsKPI1* | Sub-cellular localization |
| P24-R | ggggaccactttgtacaagaaagctgggtcttaagccttcttaaacatgac |
| P25-F | ggggacaagtttgtacaaaaaagcaggcttcatgaggaaatcaaacaccc | *CsKPI2* | Sub-cellular localization |
| P26-R | ggggaccactttgtacaagaaagctgggtcttaaaccttcatgaacatgac |
| P27-F | ggggacaagtttgtacaaaaaagcaggcttcatgaagaaaacacacactcc | *CsKPI*3 | Sub-cellular localization |
| P28-R | ggggaccactttgtacaagaaagctgggtctcagaccttcttaaacatga |
| P29-F | cagagcctggcttacacttggg | *CsKPI1* | Promoter activity |
| P30-R | accttgatatgtgggtctcacattatg |
| P31-F | cagaaagaagagtaaaccg | *CsKPI2* | Promoter activity |
| P32-R | caggtgatgtgtgttatg |
| P33-F | attaacattagtggtccatttttgacc | *CsKPI*3 | Promoter activity |
| P34-R | ctttttctctatgccataaggtctc |
| P35-F | atctggttccgcgtggatccatgaagacagcaatattcctctc | *CsKPI1* | site-directed mutagenesis |
| P36-R | ggcaaccacgccttttttag |
| P37-F | aaggcgtggttgccgtatcaactgatctg | *CsKPI1* |
| P38-R | ttaaacttctctatggcaaaccaattac |
| P39-F | gtaattggtttgccatagagaagtttaa | *CsKPI1* |
| P40-R | tcacgatgcggccgctcgagttaagccttcttaaacatgactcc |
| P41-F | atctggttccgcgtggatccatgaggaaatcaaacaccccc | *CsKPI2* | site-directed mutagenesis |
| P42-R | ggcaactacgccgtttgg |
| P43-F | aaacggcgtagttgccgtctccacagatctc | *CsKPI2* |
| P44-R | cataagcttcgatggcaaaccaatcgctc |
| P45-F | gagcgattggtttgccatcgaagcttatg | *CsKPI2* |
| P46-R | tcacgatgcggccgctcgagttaaaccttcatgaacatgactttg |
| P47-F | atctggttccgcgtggatccatgaagaaaacacacactc | *CsKPI*3 | site-directed mutagenesis |
| P48-R | tctgtggagacggcaacgacg |
| P49-F | cgtcgttgccgtctccacaga | *CsKPI*3 |
| P50-R | catacctttcgatggcaaaccaat |
| P51-F | attggtttgccatcgaaaggtatg | *CsKPI*3 |
| P52-R | tcacgatgcggccgctcgagtcagaccttcttaaacatgac |

**Supplementary Table. S4** Primers used in this study (primer application details are described in materials and methods)
